# Spatial decoupling of plaque and circulating immune programs defines a robust gene signature in atherosclerosis: A multi-cohort integrative analysis

**DOI:** 10.1097/MD.0000000000050183

**Published:** 2026-08-14

**Authors:** Ruiqiao He, Wenlin Huang, Liangqiao Hu, Yongyou Fang, Fei Yang

**Affiliations:** a Department of Cardiology, Huizhou Third People’s Hospital, Guangzhou Medical University, Huizhou, Guangdong, China; b Department of Neurology, Huizhou Third People’s Hospital, Guangzhou Medical University, Huizhou, Guangdonog, China.

**Keywords:** atherosclerosis, biomarker, peripheral blood, plaque, risk stratification

## Abstract

Atherosclerosis (AS) is driven by chronic vascular inflammation, yet the relationship between immune activity within atherosclerotic lesions and systemic immune signals in peripheral blood remains poorly defined. In this study, we performed an integrative multi-cohort analysis of gene expression profiles from atherosclerotic tissues, peripheral blood, and single-cell transcriptomic datasets to characterize spatial immune heterogeneity in AS. Differential expression and enrichment analyses revealed divergent immune-related transcriptional patterns between plaques and blood, indicating a dissociation between local and systemic immune responses. Using weighted gene co-expression network analysis and multiple machine learning approaches, we identified a 5-gene signature (with XAF1 [XIAP-associated factor 1], RNF213 [ring finger protein 213], OAS2 [2'-5'-oligoadenylate synthetase 2], and OAS3 [2'-5'-oligoadenylate synthetase 3] upregulated, and BEND5 [BEN domain containing 5]) downregulated in plaques) that robustly distinguished atherosclerotic from control samples across multiple vascular beds. This gene signature demonstrated strong diagnostic performance in tissue datasets. While its diagnostic accuracy was attenuated in peripheral blood, it retained moderate prognostic value for ischemic stroke risk, suggesting its utility as a hypothesis-generating tool for noninvasive risk stratification. Together, these findings highlight spatially distinct immune programs in AS and support the use of integrated tissue- and blood-based gene signatures for improved disease diagnosis and risk stratification.

## 1. Introduction

Atherosclerosis (AS) is a chronic, progressive disease of the arterial wall and the fundamental pathological basis of ischemic cardiovascular events, including myocardial infarction and stroke.^[1,2]^ While lipid accumulation has long been regarded as the primary driver of AS, accumulating evidence indicates that immune-mediated mechanisms critically govern plaque initiation, progression, and destabilization.^[3]^ Both innate and adaptive immune responses shape the complex cellular landscape of atherosclerotic lesions, with macrophages, T cells, B cells, and endothelial cells (ECs) coordinating pro-inflammatory and regulatory programs.^[4–6]^

Despite substantial advances in understanding local plaque biology, the relationship between vascular inflammation and systemic immune signatures remains incompletely defined. Prior studies have demonstrated that immune cells within carotid plaques exhibit a more pronounced inflammatory phenotype than their circulating counterparts, suggesting a spatial dissociation of immune activation states.^[7]^ Moreover, dynamic imbalances between pro-inflammatory/pro-thrombotic and anti-inflammatory mediators within plaques appear to critically influence disease progression. In parallel, circulating inflammatory biomarkers, such as interleukin (IL)-6 and IL-8, have emerged as potential diagnostic and prognostic indicators of AS-related disease.^[8]^ However, AS is a systemic vascular disorder that ultimately manifests across multiple arterial beds (including coronary, cerebrovascular, carotid, aortic, and peripheral arteries) rendering organ-centric prevention strategies inherently limited.^[9]^ A deeper understanding of spatially distinct immune programs is therefore essential for identifying biomarkers with robust diagnostic and prognostic relevance. However, most existing transcriptomic studies have focused on either atherosclerotic plaques or circulating immune cells in isolation, leaving the degree of coordination or dissociation between local and systemic immune programs largely unexplored.

High-throughput transcriptomic technologies, including bulk ribonucleic acid (RNA) sequencing and single-cell RNA sequencing (scRNA-seq), now enable unprecedented resolution of cellular and molecular heterogeneity within atherosclerotic lesions. Integrating these datasets with systems biology approaches, such as weighted gene co-expression network analysis (WGCNA) and machine learning algorithms, offers a powerful framework to delineate core gene networks and prioritize clinically actionable biomarkers.

In this study, we conducted a comprehensive multi-cohort, multi-omics analysis to delineate local versus systemic immune signatures in AS, identify plaque-associated gene modules and core diagnostic genes, and characterize their cellular localization and clinical relevance using scRNA-seq and machine learning approaches. Through this integrative strategy, we aim to define a concise, immune-centered gene signature that captures spatial immune heterogeneity in AS and provides a foundation for improved diagnosis and risk stratification.

## 2. Materials and methods

### 2.1. Data sources and study design

Publicly available transcriptomic datasets, including GSE43292,^[10]^ GSE202625,^[11]^ GSE57691,^[12]^ GSE100927,^[13]^ GSE28829,^[14]^ GSE41571,^[15]^ GSE242046,^[16]^ GSE21545,^[17]^ and GSE159677,^[18]^ were obtained from the gene expression omnibus database (https://www.ncbi.nlm.nih.gov/geo/).

GSE43292 served as the tissue-based discovery cohort and included 32 AS plaque samples and 32 paired macroscopically intact arterial tissue samples from patients undergoing carotid endarterectomy. This dataset was used to identify AS-associated differentially expressed genes (DEGs).

GSE202625 was used as the blood-based discovery cohort and comprised 27 peripheral blood samples from patients with coronary artery disease (CAD) and 25 samples from healthy controls. This dataset was analyzed to evaluate the detectability and translational potential of tissue-derived diagnostic gene signatures in circulation.

To validate the robustness and generalizability of the identified gene signatures in distinguishing AS lesions from non-atherosclerotic controls, 2 independent tissue-based transcriptomic datasets were analyzed. GSE57691 included 9 aortic samples from patients with aortic occlusive disease and 10 control aortic specimens from organ donors, while GSE100927 comprised 69 AS peripheral artery tissue samples and 35 control vascular tissue samples.

To further assess whether the identified gene signatures reflected intraplaque heterogeneity and disease severity, GSE28829 (16 advanced and 13 early AS plaque samples) and GSE41571 (5 ruptured and 6 stable plaques) were analyzed to evaluate expression patterns across different plaque stages and phenotypic states.

For blood-based external validation, GSE242046, which included 30 peripheral blood samples from patients with CAD and 20 control samples, was used to assess predictive performance in an independent cohort. In addition, GSE21545, comprising 97 peripheral blood mononuclear cell (PBMC) samples and 126 vascular tissue samples from patients undergoing endarterectomy, was included to evaluate whether the identified gene signatures in blood or tissue could predict the occurrence of ischemic stroke.

Finally, scRNA-seq dataset GSE159677, consisting of 3 calcified atherosclerotic core (AC) plaques and 3 patient-matched proximal adjacent (PA) tissues, was used to characterize the cellular localization and expression patterns of the identified diagnostic genes at single-cell resolution.

### 2.2. Identification of DEGs

DEGs were identified using DESeq2 (v1.40.2) for RNA sequencing data and the limma package (v3.56.2) for microarray data. Genes with an absolute log_2_ fold change (|log_2_FC|) > 0.5 and a *P* value < .05 were considered statistically significant. Volcano plots were generated using the ggplot2 package (v3.5.1) to visualize DEG distributions.

### 2.3. Gene set enrichment analysis (GSEA)

GSEA was performed to identify activated or suppressed gene ontology (GO) biological process pathways in blood or tissue samples from patients with AS. GSEA was conducted using the clusterProfiler package (v4.8.3), and enrichment results were visualized with the GseaVis package (v0.1.0).

### 2.4. WGCNA

WGCNA was performed to investigate associations between gene expression patterns and AS plaque status using the WGCNA R package (v1.72–1). ^[19]^ Lowly expressed genes were filtered to reduce background noise, and genes with a coefficient of variation > 0.1 were retained. An adjacency matrix was constructed using a soft-thresholding power (*β*) of 16 based on Pearson correlation coefficients. The adjacency matrix was transformed into a topological overlap matrix, and the corresponding dissimilarity matrix (1 − topological overlap matrix) was calculated. Hierarchical clustering was performed to identify gene modules with similar expression profiles using the parameters minModuleSize = 30 and mergeCutHeight = 0.25. Modules with an absolute correlation coefficient |*r*| > 0.5 and a *P* value < .05 were considered significantly associated with AS plaques.

### 2.5. Identification of key genes and protein–protein interaction (PPI) network construction

Key genes were identified by intersecting 3 gene sets: DEGs between AS plaques and paired intact arterial tissues in the tissue-based discovery cohort, DEGs between AS patients and healthy controls, and genes within WGCNA modules significantly associated with AS plaques. The overlapping genes were defined as AS-related key genes.

PPI networks were constructed using the Search Tool for the Retrieval of Interacting Genes/Proteins database (https://cn.string-db.org/), followed by GO and disease–gene associations enrichment analyses. Venn diagrams were generated using VENNY (v2.1.0; https://bioinfogp.cnb.csic.es/tools/venny/). PPI networks were visualized with Cytoscape (v3.10.2), and heatmaps depicting the expression patterns of key genes in blood and plaque tissues were generated using the pheatmap package (v1.0.12).

### 2.6. Machine learning–based feature selection

A total of 36 AS-related key genes were included as candidate variables for feature selection using 3 machine learning algorithms: least absolute shrinkage and selection operator (LASSO), support vector machine–recursive feature elimination (SVM–RFE), and random forest (RF). LASSO analysis was performed using the glmnet package (v4.1–8), SVM–RFE was implemented with the caret package (v6.0–94), and RF analysis was conducted using the randomForest package (v4.7–1.1) in R. Genes consistently selected by all 3 algorithms were defined as core diagnostic genes. The diagnostic performance of the identified core genes was evaluated by receiver operating characteristic (ROC) curve analysis using the pROC package (v1.18.4), and the area under the curve (AUC) was calculated to assess discriminatory accuracy. Prior to model training, gene expression matrices were log-normalized and scaled to ensure comparability across different samples. The final logistic regression model was constructed using the expression levels of the 5 core genes, and performance was evaluated using AUC alongside 95% confidence intervals (CIs). It should be noted that the feature selection and regression model development were performed on the same discovery cohorts, and these models were subsequently evaluated on external validation sets.

### 2.7. Risk score construction and ischemic event analysis

A risk score was calculated using the coefficients derived from the LASSO model based on standardized gene expression levels, according to the following formula:

Risk score = 1.0434 × XAF1 (XIAP-associated factor 1) − 0.6645 × BEND5 (BEN domain containing 5) + 0.5885 × OAS3 (2'-5'-oligoadenylate synthetase 3) + 0.5082 × RNF213 (ring finger protein 213) + 0.4824 × OAS2 (2'-5'-oligoadenylate synthetase 2).

The predictive value of the risk score for ischemic stroke was evaluated using the GSE21545 dataset. Risk scores were calculated for each sample based on the LASSO-derived model. Patients were stratified into high- and low-risk groups according to the optimal cutoff value determined using the surv_cutpoint function from the survminer package (v0.4.9), which identifies the threshold that maximizes the separation between survival curves. The occurrence of ischemic events was defined as the study endpoint. Cumulative hazard rates were estimated using the survival R package (v3.5–7), and Kaplan–Meier survival curves were generated and visualized using the survminer package (v0.4.9). Ischemic stroke was selected as the sole clinical outcome because it was the primary documented follow-up endpoint available in the GSE21545 dataset. Due to the limitations of the public metadata, adjustments for other clinical covariates (e.g., patient age, statin use, or comorbidities) could not be performed.

### 2.8. Immune infiltration analysis

Cell-type Identification by Estimating Relative Subsets of RNA Transcripts (CIBERSORT),^[20]^ a deconvolution algorithm for estimating tissue cell composition based on reference gene expression signatures, was used to characterize the immune infiltration landscape in AS plaques. The 22 immune cell-type leukocyte signature matrix gene signature matrix, comprising 547 genes that distinguish 22 human immune cell types, was applied to estimate the relative proportions of infiltrating immune cells in all samples.

For immune infiltration analyses, samples were categorized into 3 groups: control, low-risk score, and high-risk score groups, with risk stratification based on the median risk score. Differences in immune cell proportions among groups were compared, and correlations between core gene expression levels and immune cell abundances were calculated. Statistical analyses and visualizations were performed using the ggplot2 package (v3.5.1), and correlation heatmaps were generated using the pheatmap package (v1.0.12).

### 2.9. Regulatory network construction

Based on the 5 core genes, regulatory networks involving microRNA-gene and transcription factor–gene interactions were constructed using NetworkAnalyst (http://www.networkanalyst.ca).^[21]^ The resulting regulatory networks were visualized using Cytoscape (v3.10.2).

### 2.10. scRNA-seq analysis

The scRNA-seq dataset GSE159677 was processed using the Seurat R package (v5.0.1). Low-quality cells, potential doublets, and cells with more than 20% mitochondrial gene expression were excluded. Genes with low expression levels were filtered according to minimum feature thresholds. Data were log-normalized, and the top 2000 highly variable genes were selected. Following data scaling, principal component analysis was performed for dimensionality reduction. Cell clustering was conducted using the FindNeighbors and FindClusters functions, and cell types were annotated based on canonical marker genes. Batch effects were corrected using the Harmony algorithm, and residual low-quality cells were removed. Based on the 5 core genes, the AddModuleScore function was applied to calculate a risk score for each individual cell. Single-cell visualization was performed using the scop package (v0.6.0).

### 2.11. Statistical analysis

All statistical analyses were performed using R software (v4.3.1). All tests were 2-tailed, and a *P* value < .05 was considered statistically significant.

## 3. Results

### 3.1. Distinct and divergent immune signatures in atherosclerotic plaques and peripheral blood

**Figure 1.**
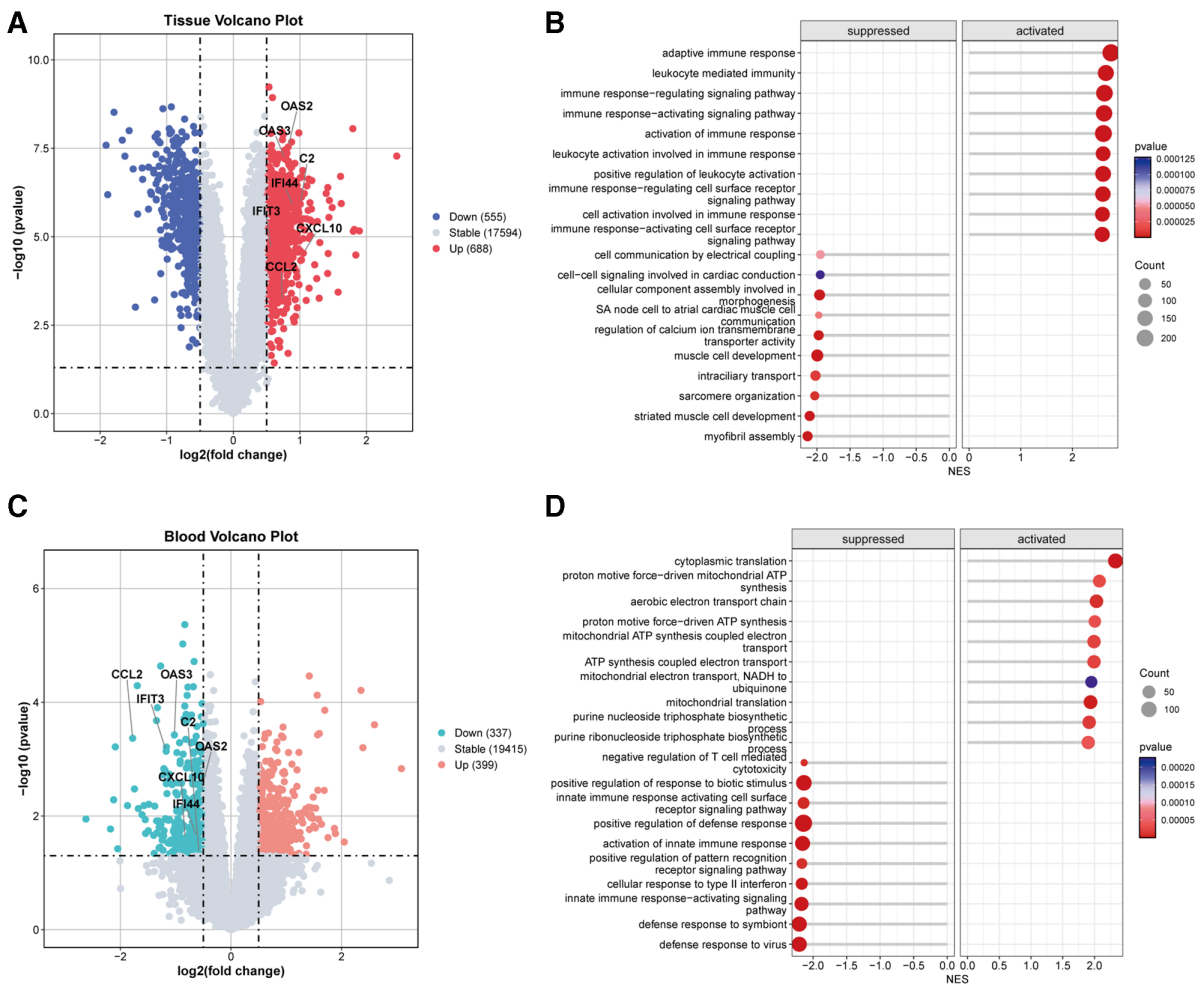
Differential gene expression and GSEA-based GO biological process enrichment in atherosclerotic tissue and peripheral blood. (A) Volcano plot showing DEGs between atherosclerotic plaques and control arterial tissues in the tissue-based discovery cohort (GSE43292), (B) GSEA of GOBP terms in atherosclerotic plaques, (C) volcano plot of DEGs between AS patients and healthy controls in the peripheral blood discovery cohort (GSE202625), (D) GSEA-based GOBP enrichment analysis of peripheral blood samples. AS = atherosclerosis, ATP = adenosine triphosphate, DEG = differentially expressed gene, GO = gene ontology, GOBP = GO biological process, GSEA = gene set enrichment analysis, NADH = reduced nicotinamide adenine dinucleotide, SA = sinoatrial.

Differential expression analysis was first performed in the tissue-based discovery cohort (GSE43292) by comparing atherosclerotic plaques with paired macroscopically intact arterial tissues. A total of 1223 DEGs were identified, including 668 upregulated and 555 downregulated genes in AS plaques. Among the upregulated genes, several interferon-stimulated genes (ISGs), such as OAS2, OAS3, interferon-induced protein 44 (IFI44), and interferon-induced protein with tetratricopeptide repeats 3, as well as inflammation- and chemotaxis-related genes involved in immune cell recruitment, including C-C motif chemokine ligand 2, C-X-C motif chemokine ligand 10, and complement C2, were prominently enriched (Fig. 1A). GSEA revealed that AS plaques were characterized by significant activation of immune-related biological processes, including adaptive immune response, leukocyte-mediated immunity, and immune response activation. In contrast, pathways associated with myofibril assembly, sarcomere organization, and muscle cell development were markedly suppressed, indicating a loss of vascular smooth muscle–related functional programs within atherosclerotic lesions (Fig. 1B).

Interestingly, an opposite transcriptomic pattern was observed in peripheral blood. Differential expression analysis of the blood-based discovery cohort (GSE202625) identified 399 upregulated and 337 downregulated genes in patients with AS compared with healthy controls. Notably, several ISGs and immune recruitment-related genes that were upregulated in AS plaques (OAS2, OAS3, IFI44, interferon-induced protein with tetratricopeptide repeats 3, C-C motif chemokine ligand 2, C-X-C motif chemokine ligand 10, and complement C2) were consistently downregulated in the peripheral blood of AS patients (Fig. 1C). Consistent with these findings, GSEA demonstrated that peripheral blood from AS patients exhibited enrichment of pathways related to cytoplasmic translation and mitochondrial translation, whereas immune-associated processes, including defense response to virus, cellular response to type II interferon, and activation of innate immune response, were significantly suppressed (Fig. 1D).

Collectively, these results reveal divergent enrichment patterns of immune-related biological processes between atherosclerotic plaques and peripheral blood, suggesting a decoupling between local lesion-specific inflammatory activation and systemic immune transcriptomic responses in AS.

### 3.2. Identification of AS-associated gene modules and key genes by WGCNA

**Figure 2.**
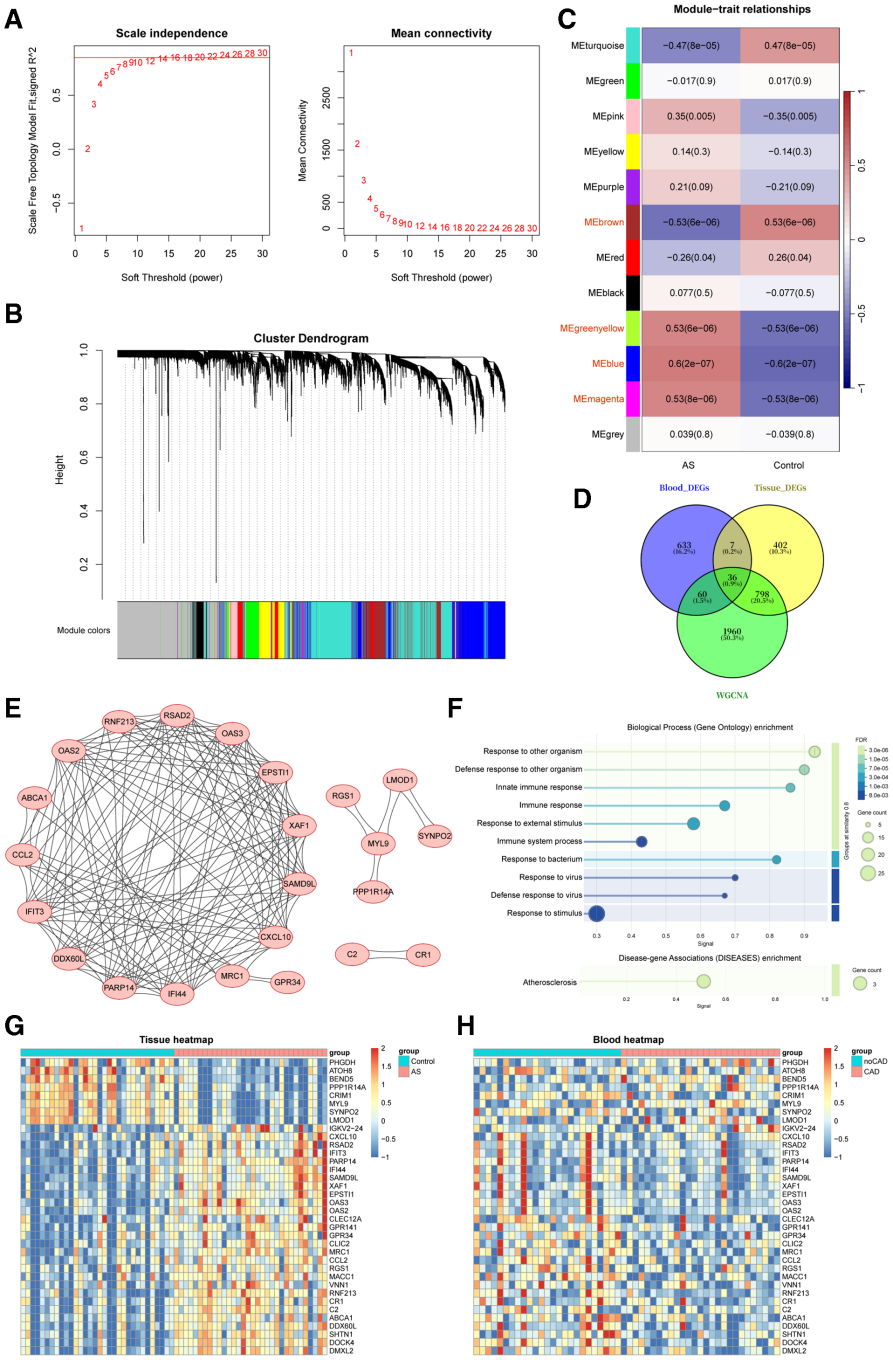
Identification of AS-associated gene modules and key genes. (A) Selection of the soft-thresholding power for WGCNA, showing a scale-free topology fit index (*R*^2^) of 0.85 at power = 16; (B) cluster dendrogram of genes based on co-expression network analysis, identifying 12 distinct modules; (C) heatmap of correlations between gene modules and AS status; (D) Venn diagram showing the overlap of DEGs from tissue and blood datasets with WGCNA module genes, identifying 36 key genes; (E) PPI network of the key genes constructed using the STRING database; (F) GOBP and DISEASES enrichment analyses of the key genes; (G and H) Heatmaps showing the expression patterns of the 36 key genes in (G) atherosclerotic tissues and (H) peripheral blood samples. AS = atherosclerosis, CAD = coronary artery disease, DEG = differentially expressed gene, DISEASES = disease–gene associations, GOBP = gene ontology biological process, PPI = protein–protein interaction, STRING = Search Tool for the Retrieval of Interacting Genes/Proteins, WGCNA = weighted gene co-expression network analysis.

To further identify genes associated with AS WGCNA was performed on the tissue-based discovery cohort. A soft-thresholding power of 16 was selected, at which the scale-free topology model fit index (*R*^2^) reached 0.85, indicating an optimal balance between network robustness and biological relevance (Fig. 2A). Based on hierarchical clustering of gene expression profiles, all genes were classified into 12 distinct co-expression modules, as shown in the cluster dendrogram (Fig. 2B). Module–trait correlation analysis (Fig. 2C) revealed that the MEbrown module was negatively correlated with AS, whereas the MEgreenyellow, MEblue, and MEmagenta modules showed significant positive correlations with AS (absolute correlation coefficient |*r*| > 0.5 and *P* < .05). The union of genes from these 4 AS-associated modules yielded a total of 2854 WGCNA-derived AS-related genes. The intersection of these module genes with DEGs identified in both tissue and peripheral blood datasets resulted in 36 AS key genes (Fig. 2D).

PPI analysis using the Search Tool for the Retrieval of Interacting Genes/Proteins database showed that 23 of the 36 key genes formed a tightly connected interaction network (Fig. 2E). GO biological process enrichment analysis indicated that these genes were mainly involved in immune-related processes, including response to other organisms, immune response, and response to bacteria. Consistently, disease–gene associations enrichment analysis highlighted a significant association with AS (Fig. 2F).

Finally, the expression patterns of the 36 key genes were visualized in tissue (Fig. 2G) and peripheral blood samples (Fig. 2H). Notably, their expression profiles further supported the previously observed decoupling between local plaque-associated transcriptional activation and systemic blood-based gene expression changes.

### 3.3. Identification of core diagnostic genes using machine learning approaches

To further refine the 36 AS-related key genes and identify the most robust diagnostic markers, 3 complementary machine learning algorithms were applied, including LASSO, SVM–RFE, and RF.

**Figure 3.**
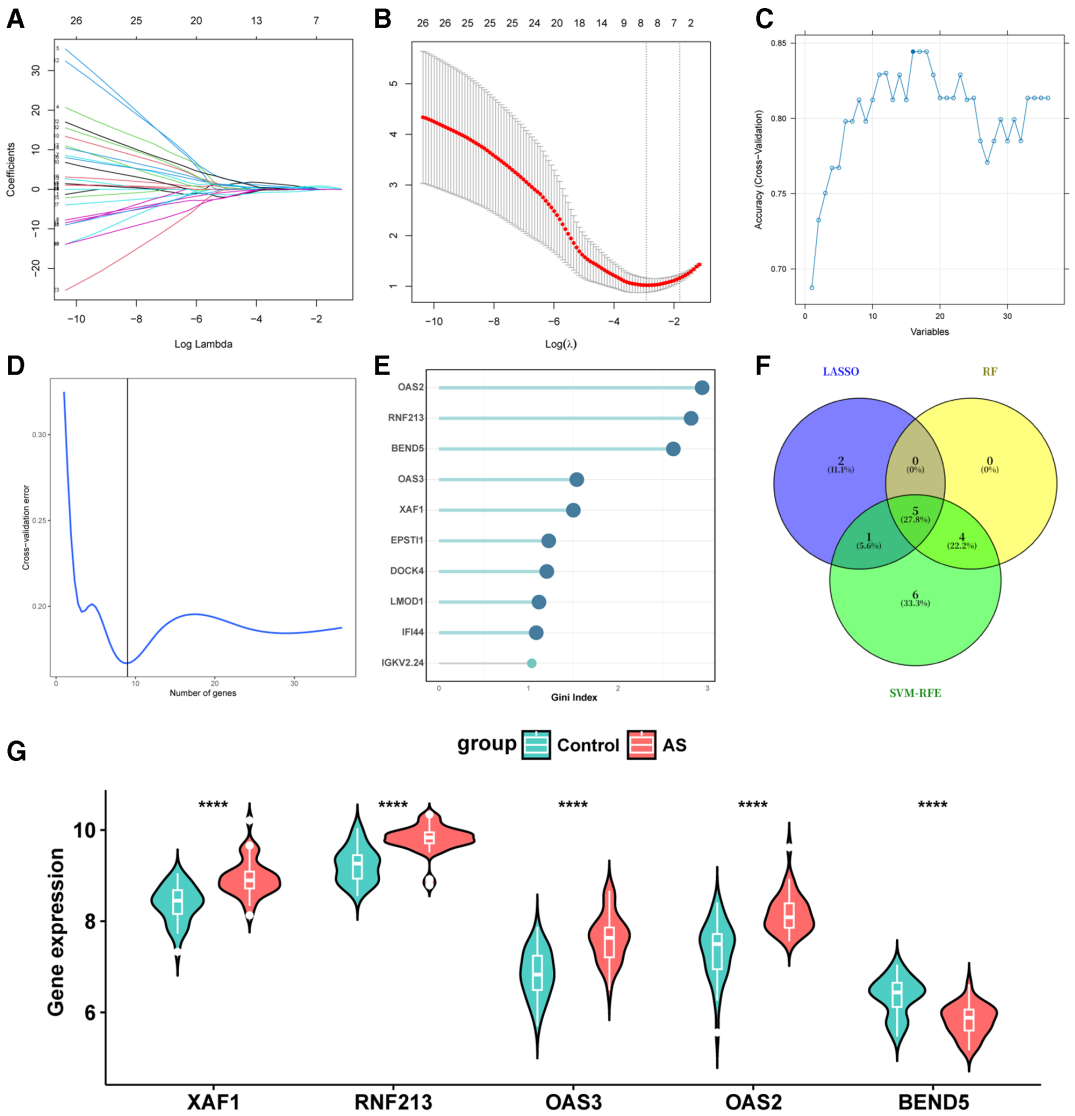
Identification of core diagnostic genes for AS using machine learning. (A and B) Feature selection using LASSO regression. Genes were selected at the optimal penalty parameter (lambda.min) determined by cross-validation; (C) gene selection using SVM–RFE, showing the highest classification accuracy at 16 variables; (D) RF analysis identifying the optimal model based on the lowest cross-validation error; (E) top 10 genes ranked by Gini importance in the RF model; (F) Venn diagram showing the overlap of genes selected by LASSO, SVM–RFE, and RF analyses, identifying 5 core diagnostic genes; (G) violin plots showing the expression levels of the 5 core genes in control and atherosclerotic plaque tissues. AS = atherosclerosis, LASSO = least absolute shrinkage and selection operator, RF = random forest, SVM–RFE = support vector machine–recursive feature elimination.

Using the LASSO regression model, the optimal penalty parameter was determined at the minimum cross-validation error (*λ* = lambda.min), resulting in the selection of 8 candidate genes (Figs. 3A and 3B). Subsequently, SVM–RFE analysis was performed to rank gene importance, and the highest classification accuracy was achieved when 16 variables were retained (Fig. 3C). In parallel, RF analysis identified the optimal model with 9 variables, corresponding to the lowest cross-validation error, and ranked genes based on the Gini importance index (Figs. 3D and 3E).

Notably, 5 genes (XAF1, RNF213, OAS3, OAS2, and BEND5) were consistently selected by all 3 machine learning methods and were therefore defined as the core diagnostic genes for AS (Fig. 3F). Expression profiling in the tissue-based discovery cohort revealed that XAF1, RNF213, OAS3, and OAS2 were significantly upregulated in AS plaques, whereas BEND5 was downregulated compared with control tissues (Fig. 3G).

### 3.4. Diagnostic performance and prognostic value of the 5-gene signature

**Figure 4.**
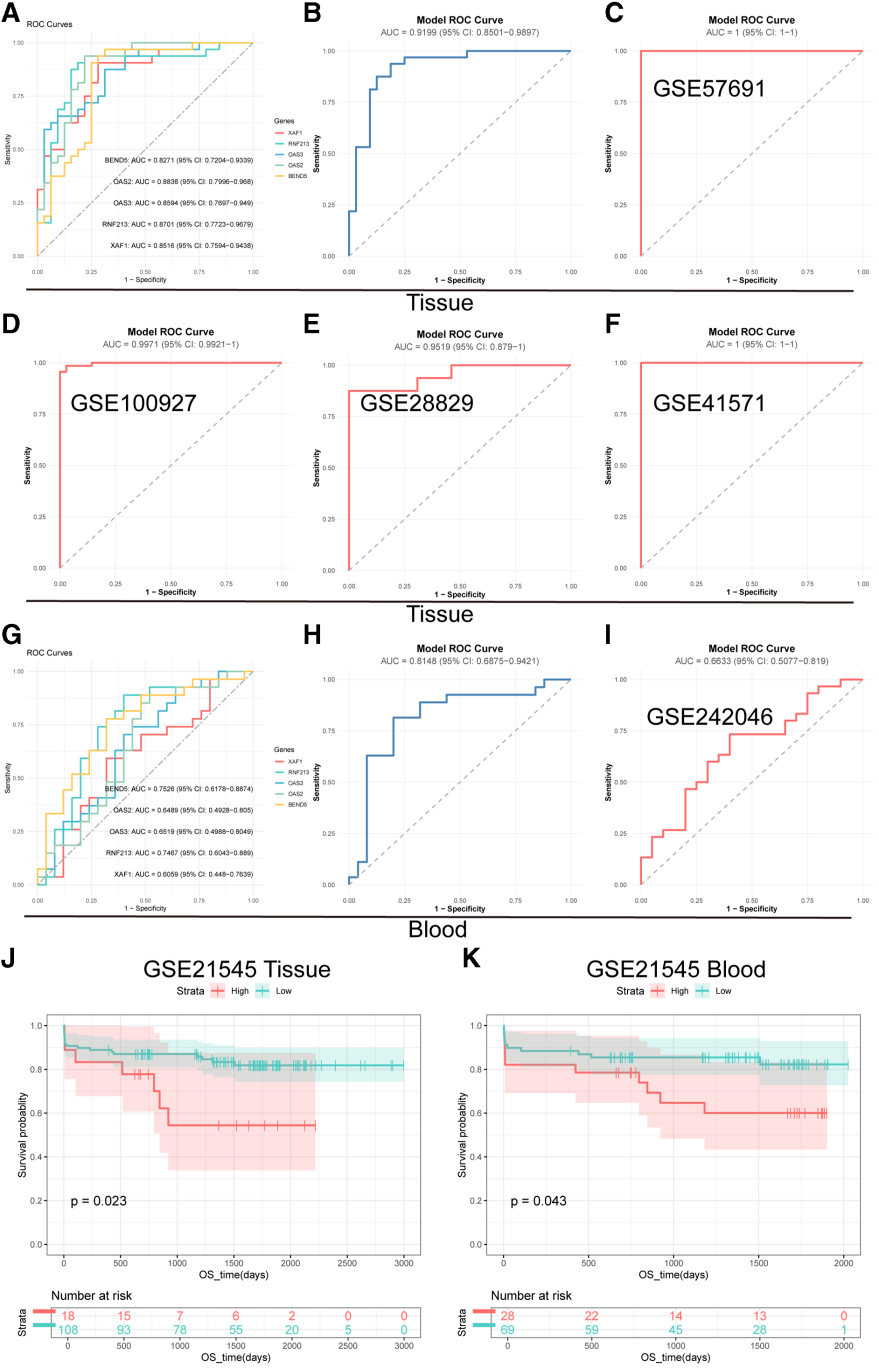
Diagnostic performance and prognostic value of the 5-gene signature. (A) ROC curves of individual genes in the tissue discovery cohort; (B) ROC curve of the 5-gene logistic regression model in the tissue discovery cohort; (C–F) ROC curves of the 5-gene model in independent tissue validation cohorts: (C) GSE57691, (D) GSE100927, (E) GSE28829, (F) and GSE41571; (G) ROC curves of individual genes in the blood discovery cohort; (H) ROC curve of the 5-gene logistic regression model in the blood discovery cohort; (I) ROC curve of the 5-gene model in the blood validation cohort GSE242046; (J and K) Kaplan–Meier curves for ischemic stroke in (J) tissue and (K) PBMC samples from GSE21545 stratified by high- and low-risk scores. AUC = area under the curve, CI = confidence interval, OS = overall survival, PBMC = peripheral blood mononuclear cell, ROC = receiver operating characterisitic.

ROC curve analyses were performed to evaluate the diagnostic performance of the 5 core genes and their combined logistic regression model. In the tissue-based discovery cohort, all 5 genes individually exhibited strong discriminatory power, with AUC values exceeding 0.80 (95% CIs are detailed in Fig. 4A). Notably, the 5-gene logistic regression model achieved an AUC of 0.9199 (95% CI: 0.8501–0.9897), indicating excellent diagnostic accuracy (Fig. 4B).

The robustness of the 5-gene signature was further validated across multiple independent AS tissue cohorts. In GSE57691, which compared aortic tissues from patients with aortic occlusive disease and controls, the 5-gene model achieved perfect diagnostic performance with an AUC of 1.00 (95% CI: 1–1) (Fig. 4C). Similarly, in GSE100927, comparing atherosclerotic peripheral arteries with control peripheral arteries, the model yielded an AUC of 0.9971 (95% CI: 0.9921–1) (Fig. 4D). In GSE28829, distinguishing advanced from early atherosclerotic plaques, the model achieved an AUC of 0.9519 (95% CI: 0.8790–1) (Fig. 4E), while in GSE41571, comparing ruptured and stable plaques, the AUC again reached 1.00 (95% CI: 1–1) (Fig. 4F).

In the blood-based discovery cohort, the individual AUC values of the 5 genes ranged from 0.6059 to 0.7526 (95% CIs are detailed in Fig. 4G). When combined into a logistic regression model, the 5-gene signature demonstrated improved diagnostic performance, with an AUC of 0.8148 (95% CI: 0.6875–0.9421) (Fig. 4H). In the independent blood validation cohort GSE242046, the 5-gene model retained moderate diagnostic capability, achieving an AUC of 0.6633 (95% CI: 0.5077–0.8190) (Fig. 4I).

To further assess the prognostic relevance of the 5-gene signature, a risk score was calculated using the LASSO-derived coefficients. Kaplan–Meier survival analyses were performed in the GSE21545 dataset, with ischemic stroke defined as the endpoint. Both AS plaque samples and AS-derived PBMC samples were analyzed separately. In both tissue (Fig. 4J) and PBMC (Fig. 4K) cohorts, patients in the high-risk group exhibited a significantly higher incidence of ischemic stroke compared with those in the low-risk group (*P* < .05).

### 3.5. Immune infiltration patterns associated with the 5-gene risk signature

**Figure 5.**
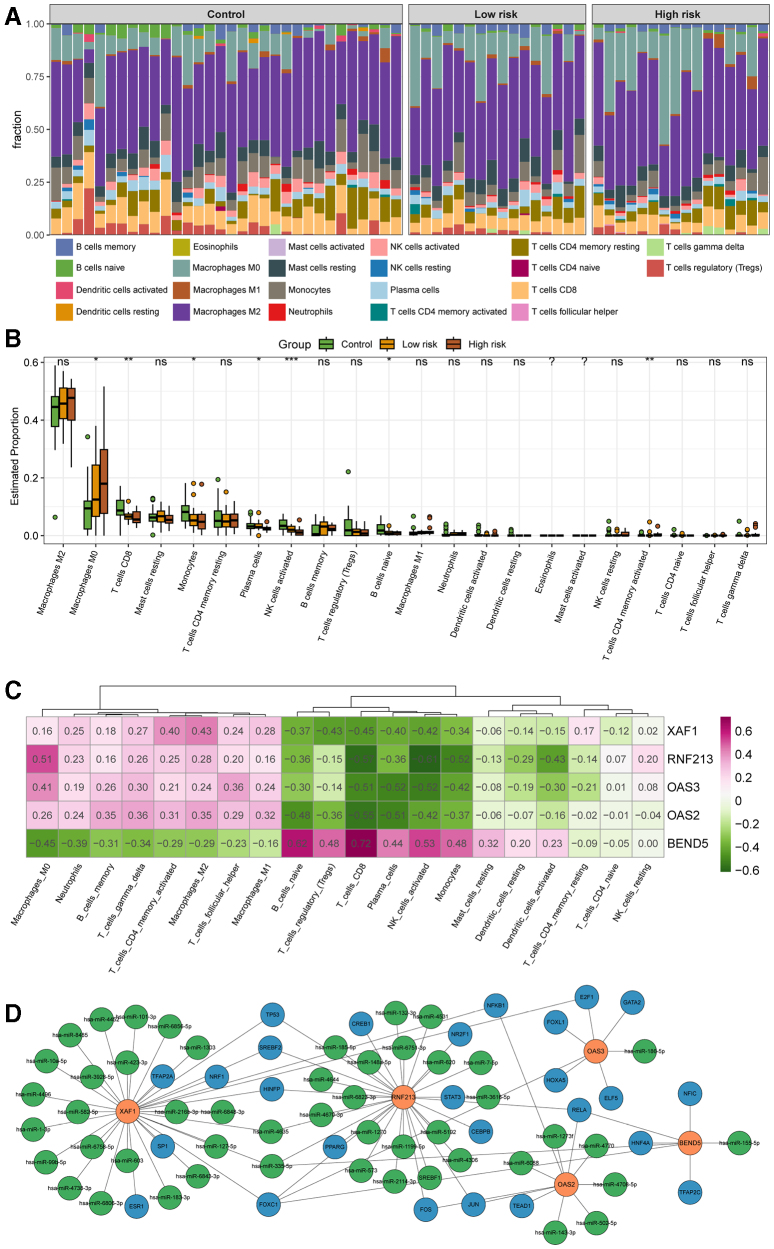
Immune infiltration landscape and regulatory networks associated with the 5-gene signature. (A) Stacked bar plot showing the relative proportions of 22 immune cell types in each sample, grouped as control, low-risk, and high-risk; (B) box plots comparing immune cell proportions among control, low-risk, and high-risk groups; (C) heatmap showing correlations between immune cell subsets and the 5 core genes; (D) miRNA-gene and TF-gene regulatory networks of the 5 core genes (orange: core genes; green: miRNAs; blue: TFs). miRNA = micro ribonucleic acid, TF = transcription factor.

To delineate the immune microenvironment associated with the 5-gene risk signature, the CIBERSORT algorithm was used to estimate the relative proportions of 22 immune cell types in each sample (Fig. 5A). Comparative analysis across control, low-risk, and high-risk groups revealed a progressive enrichment of M0 macrophages from controls to high-risk samples, whereas monocytes and activated natural killer (NK) cells exhibited a gradual decline along the same gradient (Fig. 5B).

Correlation analysis further demonstrated distinct and consistent associations between immune cell subsets and the 5 core genes. Specifically, M0, M1, and M2 macrophages, neutrophils, memory B cells, γδ T cells, activated memory cluster of differentiation (CD)4^+^ T cells, and follicular helper T cells were positively correlated with XAF1, RNF213, OAS3, and OAS2, but negatively correlated with BEND5. In contrast, naive B cells, regulatory T cells, CD8^+^ T cells, plasma cells, activated NK cells, and monocytes showed inverse correlation patterns, displaying negative associations with XAF1, RNF213, OAS3, and OAS2 and positive associations with BEND5 (Fig. 5C).

To further investigate potential upstream regulatory mechanisms, microRNA-gene and transcription factor–gene regulatory networks of the 5 core genes were constructed using NetworkAnalyst, revealing complex transcriptional and posttranscriptional regulatory interactions (Fig. 5D).

### 3.6. Single-cell localization of the 5-gene signature in atherosclerotic lesions

**Figure 6.**
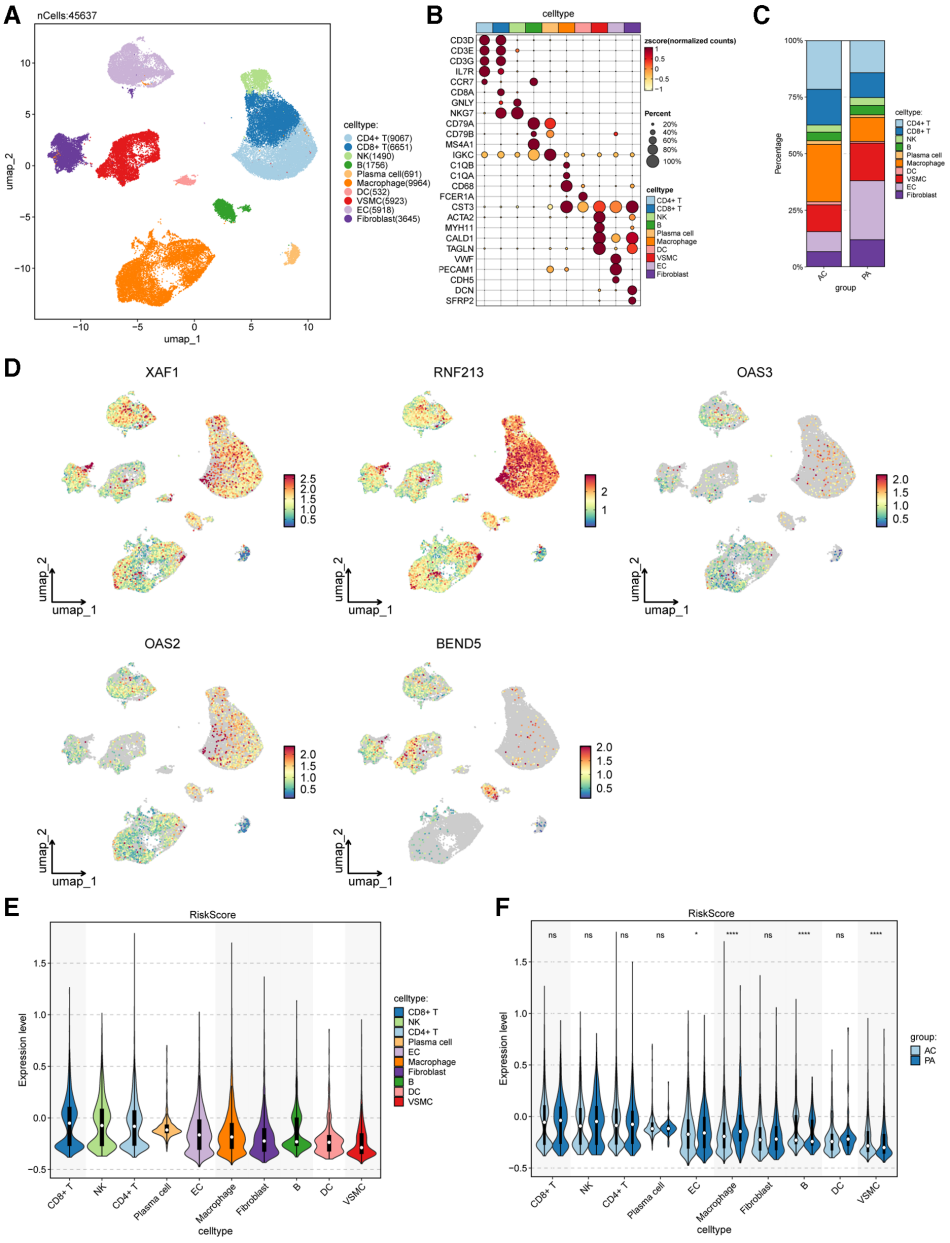
Single-cell characterization of the 5-gene signature in atherosclerotic plaques. (A) UMAP visualization of all single cells after quality control; (B) dot plot showing canonical marker gene expression for cell type annotation; (C) stacked bar plot showing cell type composition in AC and PA tissues; (D) feature plots showing the expression of the 5 core genes across cell types; (E) violin plots showing 5-gene risk scores across different cell types, ranked from high to low; (F) comparison of 5-gene risk scores between AC and PA tissues across cell types. AC = atherosclerotic core, PA = proximal adjacent, UMAP = uniform manifold approximation and projection.

To precisely delineate the cellular distribution of the 5-gene signature within atherosclerotic lesions, we analyzed a scRNA-seq dataset (GSE159677) comprising 3 calcified AC plaques and 3 patient-matched PA tissues. After stringent quality control, a total of 45,637 high-quality cells were retained for downstream analyses (Fig. 6A). Based on established canonical markers (Fig. 6B), cells were annotated into ten major cell populations, including CD4^+^ T cells, CD8^+^ T cells, NK cells, B cells, plasma cells, macrophages, dendritic cells, vascular smooth muscle cells (VSMCs), ECs, and fibroblasts. The relative cellular composition of AC and PA tissues is summarized in Figure 6C.

Gene expression profiling at single-cell resolution revealed distinct cellular localization patterns among the 5 core genes. XAF1 and RNF213 were broadly expressed across multiple immune and vascular cell types, with prominent expression in macrophages, CD4^+^ and CD8^+^ T cells, NK cells, and ECs. In contrast, OAS2 and OAS3 showed lower overall expression levels and were primarily enriched in macrophages and lymphocyte subsets, including CD4^+^ and CD8^+^ T cells and NK cells. Notably, BEND5 expression was relatively restricted, predominantly localized to B cells and VSMCs (Fig. 6D).

To further quantify the distribution of the 5-gene signature at the cellular level, a single-cell risk score was calculated for each cell based on the expression of the 5 core genes. Cell types ranked by decreasing risk scores were CD8^+^ T cells, NK cells, CD4^+^ T cells, plasma cells, ECs, macrophages, fibroblasts, B cells, dendritic cells, and VSMCs (Fig. 6E). Comparative analysis between tissue types demonstrated that ECs and macrophages exhibited significantly higher risk scores in PA tissues, whereas B cells and VSMCs displayed elevated risk scores in AC tissues (*P* < .05; Fig. 6F).

## 4. Discussion

In this study, we performed an integrative multi-cohort, multi-omics analysis to systematically characterize spatial immune heterogeneity in AS and to identify an immune-centered gene signature with diagnostic and prognostic relevance. By jointly analyzing paired vascular tissues, peripheral blood, and single-cell transcriptomes, we uncovered a pronounced dissociation between local plaque-associated immune activation and systemic immune transcriptomic responses. Through the integration of systems biology and machine learning approaches, we further defined a 5-gene signature (XAF1, RNF213, OAS2, OAS3, and BEND5) that captures key aspects of this spatial immune divergence and demonstrates consistent diagnostic and prognostic performance across independent cohorts.

A central observation of this study is the opposite enrichment of immune-related biological processes in atherosclerotic plaques and peripheral blood. While plaques exhibited robust activation of ISGs, chemokines, and leukocyte-mediated immune responses, circulating immune cells displayed relative suppression of these same pathways. This finding extends prior observations that immune cells within atherosclerotic lesions adopt a more pronounced inflammatory phenotype than their circulating counterparts.^[7]^ Rather than reflecting a uniform systemic immune activation, these data support a model of compartmentalized immune regulation, in which intense local inflammatory responses coexist with attenuated or reprogrammed systemic immune transcriptional states.

The pronounced immune divergence observed in our study must be contextualized within the broader landscape of systemic immune activation in CAD. The pathogenesis of CAD is centrally driven by endothelial activation, monocyte/macrophage recruitment, and adaptive immune dysregulation, where systemic inflammation closely interacts with local plaque vulnerability. In this context, systemic inflammatory indices and acute-phase reactants offer valuable clinical insights into patient risk beyond plaque burden alone. For instance, systemic immune-inflammatory indices have been shown to predict complications such as contrast-induced nephropathy in non-ST-segment elevation myocardial infarction cases.^[22]^ Furthermore, changes in acute-phase reactants, particularly albumin, serve as critical markers integrating inflammation, nutritional status, hepatic synthetic capacity, and overall disease severity. Recent evidence highlights the prognostic and nutritional-inflammatory implications of albumin in acute coronary syndrome,^[23]^ as well as its association with mortality and complications such as atrial fibrillation in vulnerable patient populations.^[24]^ These systemic physiological parameters underscore that while specific interferon-driven transcriptomic signatures may be attenuated in peripheral blood, the broader systemic inflammatory state remains a critical determinant of clinical outcomes. Thus, our 5-gene signature should be viewed as a complementary, hypothesis-generating tool rather than a standalone replacement for established clinical indices.

Importantly, functional immune alterations may persist in circulating cells despite the absence of overt inflammatory transcriptomic activation. A previous study demonstrated that plasma levels of IL-6 and tumor necrosis factor alpha were not significantly elevated in patients with asymptomatic carotid AS or stable CAD, whereas circulating monocytes from symptomatic patients exhibited enhanced pro-inflammatory responses upon toll-like receptor 2/4 stimulation, consistent with trained innate immunity.^[25]^ Together with our transcriptomic findings, this suggests that systemic immune quiescence at the gene expression level does not preclude functional immune dysregulation, further supporting the concept of spatial and functional immune decoupling in AS.

Among plaque-enriched pathways, interferon-related programs emerged as a dominant signal, exemplified by the consistent upregulation of OAS2, OAS3, IFI44, and IFIT family genes. Interferon signaling has been increasingly implicated in vascular inflammation, endothelial dysfunction, and plaque instability.^[26,27]^ The OAS family, comprising OAS1, OAS2, OAS3, and OASL, represents classical interferon-inducible antiviral effectors that mediate RNase L–dependent RNA degradation and are regulated by both type I and type II interferons.^[28]^ Their enrichment in atherosclerotic plaques may therefore reflect chronic endogenous nucleic acid sensing or sterile inflammatory signaling rather than acute infection.

Although interferon-associated transcriptional activation was attenuated in peripheral blood, blood-based DEGs were deliberately incorporated into the feature selection framework. This strategy was motivated by the premise that genes detectable across anatomical compartments (even when exhibiting opposing directions of regulation) may represent particularly robust and translationally relevant biomarkers. From this perspective, attenuated yet reproducible blood-based signals should not be interpreted as a limitation, but rather as a biologically meaningful manifestation of immune compartmentalization. Consistent with this notion, the 5-gene signature retained diagnostic utility in peripheral blood and demonstrated prognostic relevance for ischemic stroke, supporting previous reports that circulating immune signatures can encode clinically meaningful vascular risk information.^[9,29]^

Immune deconvolution analyses further demonstrated that increasing 5-gene risk scores were associated with a progressive enrichment of macrophage populations, particularly M0 macrophages, accompanied by a concomitant reduction in monocytes and activated NK cells. These shifts are compatible with impaired monocyte recruitment, altered macrophage differentiation trajectories, and dysfunctional innate immune surveillance during advanced stages of AS.^[30–32]^ Correlation analyses revealed divergent immune associations between BEND5 and the interferon-responsive genes XAF1, RNF213, OAS2, and OAS3. Whereas the latter were consistently associated with macrophage- and T cell-dominated inflammatory states, BEND5 exhibited positive correlations with lymphocyte subsets enriched for regulatory or cytotoxic functions, suggesting distinct immunological roles within the atherosclerotic milieu.

Single-cell transcriptomic analyses further localized the 5-gene signature to specific immune and vascular cell populations within atherosclerotic tissues. The broad expression of XAF1 and RNF213 across immune and endothelial compartments supports their potential roles as integrative nodes within plaque-associated immune networks. In contrast, although BEND5 remains poorly characterized in the context of AS, its restricted expression in B cells and VSMCs suggests a more specialized role in immune regulation or vascular homeostasis. Notably, elevated single-cell risk scores were observed not only within plaque cores but also in adjacent vascular regions, indicating the presence of subclinical immune activation beyond the lesion core and supporting the concept of spatially extended vascular inflammation.

Several limitations of this study must be acknowledged. First, the retrospective nature of the analyzed public datasets precludes causal inference. Second, the claim of divergent immune programs is based on independent cohorts rather than a formal cross-compartment comparison, as the tissue cohort (carotid plaques) and the blood cohort (CAD populations) were not paired samples from the same individuals, introducing potential confounding from different disease phenotypes. Third, the comparability of our heterogeneous validation cohorts may be affected by the use of diverse vascular beds (carotid, aortic, peripheral), different control definitions, and varying transcriptomic platforms. Fourth, regarding methodology, performing feature selection and logistic regression model development in the same discovery dataset carries a risk of overfitting and optimism, and the direct transferability of risk score coefficients across different microarray platforms requires caution. Consequently, the diagnostic and prognostic value of this signature in peripheral blood should be strictly interpreted as hypothesis-generating and necessitates prospective, resampling-based internal and external validation. Fifth, our survival analysis was restricted to ischemic stroke due to the specific endpoint documented in GSE21545, and we were unable to adjust for critical clinical covariates. Finally, CIBERSORT-derived immune proportions should be interpreted cautiously, as the 22 immune cell-type leukocyte signature matrix reference matrix was derived from peripheral blood and may not fully capture the phenotypic complexity of tissue-resident immune cells in atherosclerotic plaques.

## 5. Conclusions

In summary, this study reveals a pronounced spatial dissociation between plaque-localized and systemic immune transcriptomic programs in AS. By integrating bulk and single-cell transcriptomic data across multiple cohorts, we identify a 5-gene immune-centered signature that captures this spatial heterogeneity. While this signature demonstrates strong diagnostic performance in vascular tissues, its application in peripheral blood remains hypothesis-generating. These findings highlight the importance of spatial context in vascular immunology and provide a framework for integrating tissue- and blood-based immune biomarkers to improve risk stratification and clinical assessment in atherosclerotic disease.

## Author contributions

**Conceptualization:** Ruiqiao He, Wenlin Huang, Fei Yang.

**Data curation:** Wenlin Huang.

**Formal analysis:** Ruiqiao He.

**Investigation:** Ruiqiao He, Wenlin Huang, Liangqiao Hu, Yongyou Fang, Fei Yang.

**Project administration:** Fei Yang.

**Resources:** Wenlin Huang, Fei Yang.

**Software:** Ruiqiao He, Wenlin Huang.

**Supervision:** Fei Yang.

**Validation:** Ruiqiao He, Wenlin Huang, Liangqiao Hu, Yongyou Fang, Fei Yang.

**Visualization:** Ruiqiao He, Wenlin Huang.

**Writing – original draft:** Ruiqiao He.

**Writing – review & editing:** Ruiqiao He, Wenlin Huang, Liangqiao Hu, Yongyou Fang, Fei Yang.
